# The Foraging Tunnel System of the Namibian Desert Termite, *Baucaliotermes hainesi*

**DOI:** 10.1673/031.010.6501

**Published:** 2010-06-14

**Authors:** Walter R. Tschinkel

**Affiliations:** Department of Biological Science, Florida State University, Tallahassee, FL 32303

**Keywords:** foraging, harvester termite, nest construction, architecture, construction

## Abstract

The harvester termite, *Baucaliotermes hainesi* (Fuller) (Termitidae: Nasutitermitinae), is an endemic in southern Namibia, where it collects and eats dry grass. At the eastern, landward edge of the Namib Desert, the nests of these termites are sometimes visible above ground surface, and extend at least 60 cm below ground. The termites gain access to foraging areas through underground foraging tunnels that emanate from the nest. The looseness of the desert sand, combined with the hardness of the cemented sand tunnels allowed the use of a gasolinepowered blower and soft brushes to expose tunnels lying 5 to 15 cm below the surface. The tunnels form a complex system that radiates at least 10 to 15 m from the nest with crossconnections between major tunnels. At 50 to 75 cm intervals, the tunnels are connected to the surface by vertical risers that can be opened to gain foraging access to the surrounding area. Foraging termites rarely need to travel more than a meter on the ground surface. The tunnels swoop up and down forming high points at riser locations, and they have a complex architecture. In the center runs a smooth, raised walkway along which termites travel, and along the sides lie pockets that act as depots where foragers deposit grass pieces harvested from the surface. Presumably, these pieces are transported to the nest by a second group of termites. There are also several structures that seem to act as vertical highways to greater depths, possibly even to moist soil. A census of a single nest revealed about 45,000 termites, of which 71% were workers, 9% soldiers and 6% neotenic supplementary reproductives. The nest consisted of a hard outer “carapace” of cemented sand, with a central living space of smooth, sweeping arches and surfaces. A second species of termite, *Promirotermes* sp. nested in the outer carapace.

## Introduction

Termites can be roughly grouped into those species that nest within their food, usually wood, and those that nest elsewhere and must leave their nest in order to forage for food. Of the latter type, nests may be arboreal or subterranean, centrally located or dispersed into small, connected units. Most termites shun the open air, and travel to and from the foraging area by way of subterranean tunnels or covered galleries. Many species also cover the foraged material with sheet galleries before dining.

Among ground-nesting termites, nests may be hidden below ground, or they may be conspicuous features of the landscape, such as the mounds of the southern African species of *Macrotermes* or *Trinervitermes*. Given a central nest, the need to forage for food and an aversion toward open air, it is obvious that many termites must create subterranean foraging tunnel systems. Such systems, however, have rarely been studied, and are usually hardly mentioned (if at all) in reviews of termite biology. Even an authoritative treatment, such as Noirot's (1970) review of the nests of termites, gives short shrift to how termites travel from their nests to their foraging areas. Typically, it is assumed that the termites travel in subterranean foraging tunnels (e.g. Sands 1961), and indeed, the few existing studies of subterranean foraging tunnels have revealed tunnel systems of remarkable size and scale (Howse 1970; reviewed by Lee and Wood 1971). Most mound-building species exit their nests through subterranean foraging tunnels that run a few cm below the surface. In some species, the tunnels are short, and the termites travel some distance on the ground surface, but in others, the tunnels may extend 25 to 30 m (or even 60 m) from the mound. For example, the Australian termites *Coptotermes lacteus, C. brunneus, C. acinaciformis* and *Nasutitermes exitiosus* constructed systems with 9 to 30 tunnels emanating from the mound and extending 25 to 30 m to the dead wood on which the termites were feeding (Ratcliffe and Greaves 1940; Hill 1942; Greaves 1962). In *C. lacteus*, tunnels were more or less radial, with few cross connections, but with shafts to deeper soil. In *N. exitiosus*, the radial tunnels were cross-connected. Hill (1925) noted subterranean passages with flattened lumena thickly floored with “rejectamenta” radiating outward from a nest of the Australian *Mastotermes darwiniensis*, but he did not trace these passages far. A particularly thorough study is that of Darlington (1982), in which the underground foraging passages of *Macrotermes michaelsoni* were exposed and quantified.

Many termites do not build mounds that show above ground, but construct entirely subterranean nests, with tunnels to the surface. The African harvester termite, *Hodotermes mossambicus*, is well studied because of occasional subterranean encounters during the digging of trenches for construction (Hartwig 1963, 1965; Coaton and Sheasby 1975). These encounters revealed that nests are located an average about 1.4 m below ground, but can be as shallow as a few cm or as deep as 6.7 m. Large passages connect these subterranean nests to each other, and smaller passages give the termites access to the surface where they dump excavated soil and forage for grass. Foraged grass is first placed into small, superficial chambers for later transportion to the nests and consumption.

None of the reports on subterranean gallery systems describe architectural details of the tunnels themselves or how they are constructed. This paper reveals the intricate and subtle architecture of the foraging tunnels of the Namibian harvester termite, *Baucaliotermes hainesi* (Fuller) (Termitidae: Nasutitermitinae), and describes how this complex system probably serves the foraging needs of the termites. Like other harvester termites, *B. hainesi* foragers cut pieces of grass on the ground surface, and carry these back to their nest. The range of this species is limited to southern Namibia and the northwestern Cape Province of South Africa (Coaton and Sheasby 1973).

## Materials and Methods

### The study site

The study site was located at latititude - 24.9702, longitude 15.9323 (according to Google Earth) in the NamibRand Nature Reserve, a private reserve of about 180,000 ha. The soil was red sand largely stabilized by the grasses, *Stipagrostis uniplumis* (Licht) De Winter and *S. giessii* Kers (Poales: Poaceae), with circular, bare areas 5 to 15 m in diameter termed “fairy circles” (van Rooyen et al. 2004), and abundant animal trails crossing it in multiple directions. The site sloped gently from about 1100 m elevation at the base of Jagkop mountain to about 940 m just short of the Bushman Hills. Our two excavations were at approximately 1085 to 1090 m elevation. This area has an arid climate where rainfall averages between 50 and 150 mm per annum but is highly variable from year to year.

**Figure 1.**
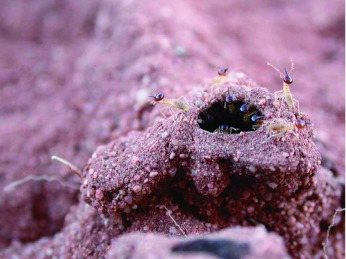
Tunnels in current use by the termites could usually be recognized by the termites found within them. Here, nasute soldiers are defending a broken tunnel. High quality figures are available online.

### Tunnel excavation and mapping

Nests of *B. hainesi* were regularly visible at the surface as small mounds of cemented material 10 to 15 cm high. All excavation work was completed between October 22 and November 3, 2007. Tunnels were initially exposed by trenching around the nest to locate tunnels, and excavated outward from there. The looseness of the dry sand, combined with the relative hardness of the cemented sand tunnels facilitated exposure of the tunnels. The sand over the tunnels was loosened with a soft hand broom, and the loosened sand was blown away with a gasoline-powered lawn blower (Husqvarna Model 356 BTx) (Video 1, 2, available online). This process produced a shallow trench 10–15 cm deep with the mostly intact tunnels in the bottom. Branches and intersections were sometimes followed, but, for many branches, only the initial few cm were exposed, leaving an unknown but substantial fraction of the entire tunnel system unexposed. Tunnels that were in use were distinguished from abandoned tunnels because the former remained intact upon excavation and/or contained live termites when broken (Figure 1). Abandoned tunnels tended to break, and were often filled with sand.

In this manner, large parts of the foraging tunnel systems of two focal nests were exposed, one located at (lat, long) - 24.96960, 15.93284 and the second at - 24.96981, 15.93403. The exposed tunnel systems were mapped by making a series of overlapping digital photographs (with a scale) from a uniform height (∼ 1 m), like aerial photographs, and then combining these into a photomosaic. A scale map was then made from each photomosaic.

### Nest census

Before exposing the second tunnel system, the nest was excavated for census. The nest was carefully broken into pieces, beginning at the top, and all live termites, as well as grass pieces, were collected by aspiration and preserved in alcohol for later counting.

**Video 1.**
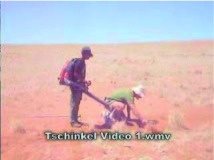


**Video 2.**
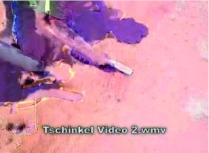


The dissection and collection took two days. Termites from the mound and each quarter of the nest from the top downwards were preserved separately. A sample of 100 each of workers, soldiers and neotenic supplementary reproductives (there were no mature alates in the nest) were killed by freezing and air-dried for later determination of dry weight.

Counts were carried out in the laboratory at Florida State University. The alcohol was drained off, and the total weight of (wet) termites from each nest portion was determined. Haphazard subsamples were then taken, weighed and the termites of each type counted. Multiplying these counts by the factor = (total weight/sample weight) gave estimates for each nest quarter, and the sum of these gave the total for the nest.

## Results

**Figure 2.**
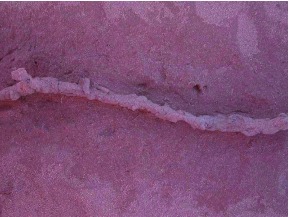
Brushing with a soft hand broom and blowing the sand away exposed the foraging tunnels a few cm below the ground surface. These tunnels were constructed of cemented sand and retained their structure despite brushing and blowing. High quality figures are available online.

The brushing and blowing removed the semi-aggregated sand overburden to expose tunnels whose walls retained their integrity because they were constructed of cemented sand (Figure 2). The tunnels are thus not simply hollows excavated in the sand, but have walls reinforced with what can be seen as “termite concrete” (which is of an unknown nature). Although the tunnels broke upon rough handling, with care, sections could also be removed for closer inspection, transport and photography.

### Tunnel architecture

**Figure 3.**
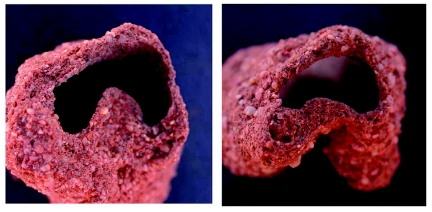
Cross sections of tunnels almost always showed a raised central portion, as seen in these two representative views. The raised central portion was the highway on which the termites traveled, while the pocket to the sides served as temporary depots for foraged grass pieces. High quality figures are available online.

**Figure 4.**
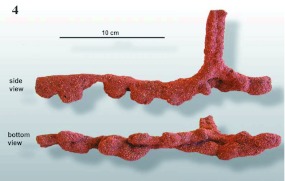
Cleaned of all loose sand, tunnels always displayed a lumpy appearance, as in this representative section, viewed both from the side and from below. The vertical extension in the side view is a riser opening to the ground surface. High quality figures are available online.

**Figure 5.**
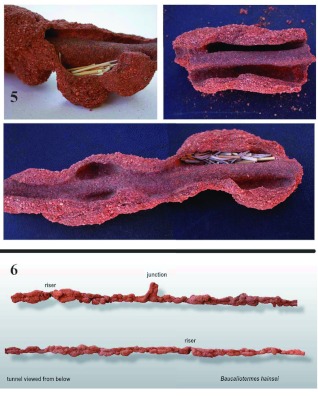
When the lumps on the tunnels were opened, they revealed lateral pockets that often contained grass pieces and served as temporary depots for grass foraged from the surface and awaiting transport to the nest. **Figure 6.** A longer section of tunnel viewed from below, showing the consistently lumpy structure. The branch in the top section is not a riser but a junction with another tunnel. Tunnels typically narrow briefly where they emit risers to the surface (not visible in this view from below). High quality figures are available online.

Tunnel architecture was complex. In cross section, most tunnels showed a raised central portion with deep pockets along both sides (Figure 3). Sections of tunnels freed of loose sand were rarely simple tubes, but showed many bulges and bumps on their undersides (Figure 4). Careful dissection of tunnels and bumps showed that the raised, central portion was a smooth roadway that ran the length of all tunnels, probably serving as the main travel path for the termites in the tunnels. Along both sides of this roadway were pockets of varying depth and geometry (Figure 5). Many of these contained pieces of grass harvested from the surface by foragers, so it is reasonable to presume that the pockets serve as temporary depots for harvested grass waiting to be transported nestward, possibly by a different group of termites than the group that harvested the grass. (Figure 6 shows a view of the underside of approximately 1 m of tunnel.

**Figure 7.**
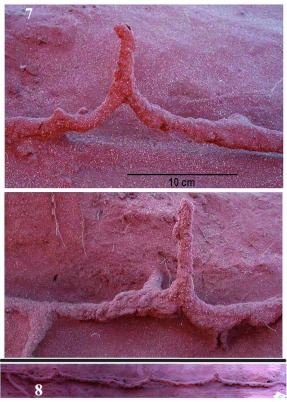
Two examples of risers that connect the tunnels to the surface. Risers were always associated with an upward swoop of the tunnel. The lower view also shows two junctions with other tunnels and one tunnel descending to greater depths. The risers were typically closed when the termites were not foraging. **Figure 8.** A section of tunnel almost a meter long, showing that risers were typically associated with the upward swoop of the tunnels. Most of the height of the risers has been broken off in this view. High quality figures are available online.

Termites in the tunnels could gain access to the surface through vertical risers, 5 to 15 cm in height, that could be opened to the surface (Figures 4 and 7). Riser openings were usually closed during the day, as this termite species forages mostly at night. Risers were very fragile, and it required great care during excavation to keep them intact. In most images, the former location of risers is seen as a double opening because two upward legs of the tunnel broke below their point of junction. The distance between risers averaged 50 to 75 cm among tunnels (SD 14 to 40 cm), suggesting that the termites rarely needed to travel more than one half to one meter on the surface.

The tunnels did not run a uniform depth below the surface, but swooped up and down between the risers, with the high points at the riser junctions and the low points about midway between risers (Figure 8). The internal runway therefore had “a roller coaster” or wave geometry. Measured from the riser-tunnel connection to the lowest upper tunnel surface between risers, the tunnel dip averaged 5 to 8 cm among tunnels, with a standard deviation of 1.5 to 2 cm. One dip was 21 cm, but the significance of this large deviation was unclear.

**Figure 9.**
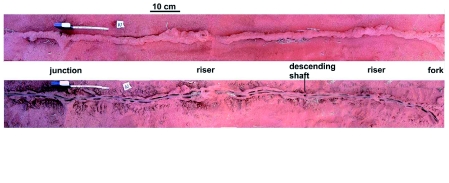
A section of about 3.4 m of tunnel (upper) from which the roof has been removed (lower), exposing the regular disposition of lateral depots and the central runway on which the termites probably travel. Grass was more commonly found in depots near risers, but these tunnels had been disconnected from the central nest for two days, so the distribution of forage may not be representative of normality. High quality figures are available online.

Careful wetting of the upper surface of exposed sections of tunnel allowed for removal of the tunnel roof to expose the depot and riser structure of two approximately two-meter-long sections (Figure 9). The upper image shows the tunnel before removal of the roof, and the lower, after. Depots can be seen along the entire length on both sides and were most likely to contain grass adjacent to risers.

One section also contained a tunnel that descended to greater depth.

**Figure 10.**
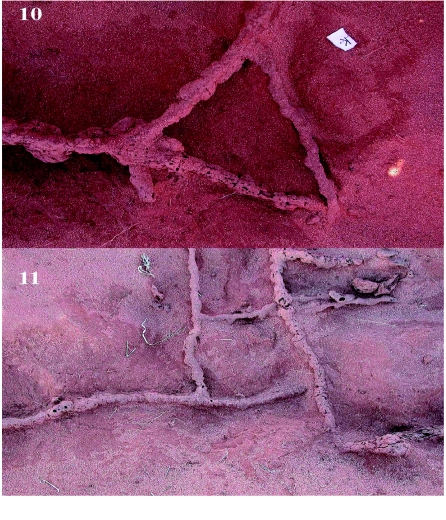
Perpendicular intersections often showed cut-offs that shortened the travel distance. **Figure 11.** Near the nests, foraging tunnels often showed rectilinear arrangements. High quality figures are available online.

Tunnels frequently intersected or branched, sometimes in rather complex configurations. Cut-offs that shortened travel distance at more or less-perpendicular intersections were common (Figure 10). Near the nests, tunnel intersections tended to form rectilinear grids (Figure 11). Occasionally, tunnels crossed without joining, a termite version of a fly-over.

### The tunnel systems

**Figure 12.**
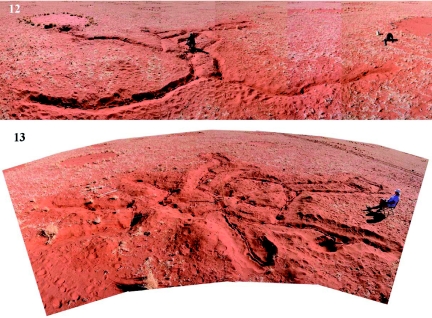
A 120° panoramic view of the first excavation. The exposed tunnels lie in the trenches, and Paul is indicating the location of the nest. The tunnels contacted a fairy circle in the center, and passed through one at the right. The total length of tunnels was about 76 meters, and the area enclosed by them about 110 m^2^. **Figure 13.** A 120° panoramic view of the second excavation. The nest was at the center of this excavation, but was removed for analysis before the tunnels were exposed. The white square at the upper left is a 1 m^2^ sampling device. High quality figures are available online.

**Figure 14.**
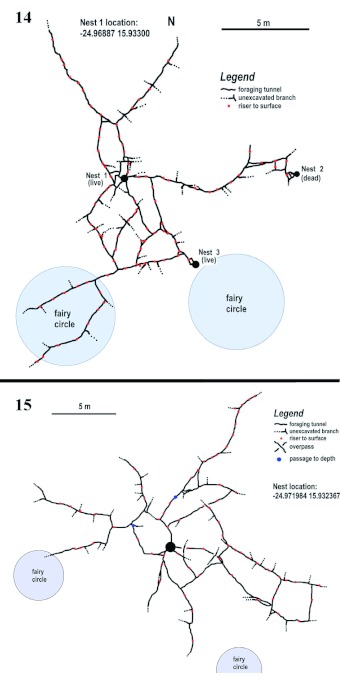
A scale map of the first excavation, showing tunnel locations, nest location and the relationship to two fairy circles. Dotted lines indicate unexcavated branches and red dots show the locations of risers to the surface. This system connected two live and one dead colony. **Figure 15.** A scale map of the second excavation. Coding similar to Figure 16, with the addition of the blue dots showing where tunnels descended to greater depth. High quality figures are available online.

Over the course of several days, large parts of the tunnel systems of the two focal nests were exposed . The total length of tunnels exposed was 76 m within an area of roughly 170 m^2^ in the first excavation and 110 m of tunnel in an area of about 300 m^2^ in the second excavation. Figures 12 and 13 show an approximately 120° panorama of each of these and reveal the scale of the termite enterprise. The exposed foraging tunnels lie in the bottom of the trenches visible in the images. Maps created from the photomosaics of these excavations are shown in Figures 14 and 15. These reveal several key features: (1) the tunnels tend generally to radiate outward from the nest; (2) the many unexcavated side-branches suggest that the area is actually underlain by a dense network of intersecting tunnels, with no area more than a meter or so from a tunnel; (3) the frequent placement of risers to the surface means that the foraging area of the termites is more or less saturated with access points, and that the termites need travel only short distances on the ground surface; (4) the tunnels probably extend outward much farther than was excavated (there was no evidence that the tunnels ended where we stopped excavating); (5) the tunnels in the first excavation (Figure 14) connected two live and one abandoned nest, suggesting that colonies of this termite may occupy more than one nest, and that nests are sometimes abandoned; (6) connected nests also suggests that the entire suitable habitat may be underlain by a network of foraging tunnels.

### Access to deeper soil

**Figure 16.**
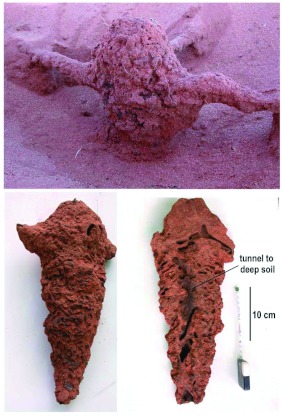
A structure in line with a foraging tunnel connecting a horizontal tunnel to one descending to greater depth. The termites almost certainly had tunnels at least deep enough to reach damp soil. High quality figures are available online.

**Figure 17.**
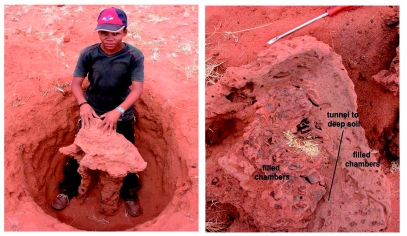
What at first appeared to be a nest was shown by excavation and dissection to be an abandoned nest being used as a vertical tunnel to greater depth. Most of the former nest chambers had been filled with sand. Note that the termites have constructed a second, parallel tunnel to depth. High quality figures are available online.

In the second excavation, two structures looked like small subsidiary nests located along the tunnel system. Dissection showed them not to be nests, but rather large, vertical tunnels that seemed to descend to deeper soil (Figure 16). The tunnel was not excavated below about 0.5 m, but there was no sign that the tunnel direction changed. The second nest mound that was excavated turned out to be an abandoned nest that was being used as a vertical tunnel to deeper soil (Figure 17).

In this case, the termites had also constructed a narrower tunnel to deep soil next to the abandoned nest. Most of the chambers in this abandoned nest had been filled with sand, and only the central core was being used as a vertical tunnel, along with the purpose-built tunnel next to the former nest.

### Dissection and census of a nest

**Figure 18.**
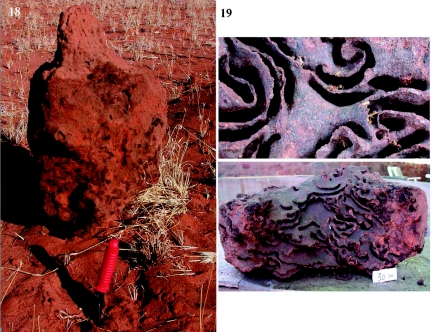
The nest from excavation 2, removed from the soil and ready for dissection. The mound on top was all that was exposed above ground level. Approximately 50 cm lay below ground. **Figure 19.** The internal structure of the nest consisted of swooping arches and surfaces at a fairly constant distance apart and composed of a dark, smooth material (probably sand and termite excreta). Note the thick “carapace” surrounding the living space in the bottom view.High quality figures are available online.

Before exposing the second tunnel system, the focal nest (Figure 18) was excavated for dissection and census of the contained termites. The nest was constructed of a hard outer “carapace” of cemented sand and filled chambers, and an interior living space of sweeping surfaces and arches of a dark, smooth material (stercoral carton), with fairly constant spacing between surfaces (Figure 19).

The nest was home to about 45,000 termites, of which about 6,000 (13%) were immature (but very small immatures, and eggs were not counted), 32,300 (71%; 36 g, dry) were workers and 4,100 (9.1%; 3 g, dry) were soldiers. In addition, there were about 2,800 (6.2%; 5 g, dry) immature reproductives with wing buds. No primary reproductives and no “royal cell” were found, suggesting this may have been a subsidiary nest (or calie).

The termites were not evenly distributed within the nest. The above-ground mound contained very few termites. About 63% of the termites were found in the second and third quarters of the nest, that is, the center or core, with only about 3% in the top quarter and 13% in the bottom quarter. However, because it took two days to dissect the nest, this distribution does not necessarily represent the natural distribution.

**Figure 20.**
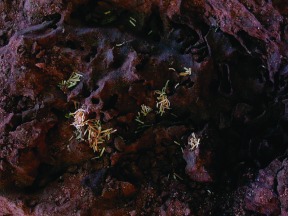
Abundant grass pieces were found in the second quarter of the nest, but the third quarter contained much less and the bottom almost none. Grass pieces entered the nest through connections with foraging tunnels about 10–15 cm below ground. High quality figures are available online.

A great deal of grass was found in the nest (Figure 20), but these grass clippings were not evenly distributed. The top quartile (0– 10 cm) contained 3.6 g of grass, the next 10.5 g, the third 0.75 g and the bottom almost none. This distribution is probably the result of the depth at which the nest connects to the foraging tunnels, about 10– 15 cm below ground, combined with the consumption of the grass as it is moved deeper toward the core of the nest where the bulk of the termites were located. The total dry weight of grass in the nest was about 15 g, probably a small fraction of what was still in the tunnel system depots.

### *Promirotermes* sp.

A species of smaller termite, *Promirotermes* sp., was found co-nesting with *B. hainesi*. Several chambers containing workers, soldiers and reproductives were located in the carapace surrounding the main *B. hainesi* nest. The relationship of this species to *B. hainesi*, the “host,” is unknown.

## Discussion

Like many other species of termites, *B. hainesi* operates on an impressive scale. Workers from each nest travel to and fro in the foraging tunnel system, harvesting grass from at least several hundred square meters.

The scale of this endeavor is of similar magnitude as several other species of mound-building termites, including *C. lacteus, C. brunneus, C. acinaciformis, N. exitiosus* (Ratcliffe and Greaves 1940; Hill 1942; Greaves 1962) and *M. michaelsoni* (Darlington 1982). Lee and Wood (1971) suggest that the underground foraging networks of subterranean termites are probably of great ecological importance. No one who has been to Africa or Australia could argue with that claim.

Previous reports on subterranean foraging tunnels gave few architectural details of their construction. The only two exceptions are Greaves (1962), who reported that the tunnels of *C. acinaciformis*, a wood-feeding species, were made of cemented soil with a simple, flattened lumen in which the termites traveled, and Darlington (1982), who described part of the foraging passage system of the fungus-gardening *M. michaelsoni* in great quantitative detail. The aeolian sands of the Namib Desert were ideal for exposing architectural details because the surrounding sand could be loosened with a soft brush and blown away, but the cemented sand that formed the tunnels remained intact, revealing subtle, complex and functional architecture. Such discrimination would have been difficult in more compacted or fine-grained soils.

Ironically, one of the clearest recent exposures of a subterranean termite tunnel system involved fossil termite nests dating to the upper Miocene and Pliocene eras (3–7 million year ago) in Chad (Duringer et al. 2007). These fossils were attributed to an ancestral fungus gardening Macrotermitinae, and they consisted of many small globular nests connected by rectilinear side tunnels to a straight main tunnel up to tens of meters long. The entire network of tunnels and chambers was all in a plane, with no evidence of vertical connections. In this regard, the layout seems somewhat similar to the nest arenas of the fungus gardening *Odontotermes fulleri* in which all chambers were located less than 30 cm below the surface (Darlington 2007).

Depots for foraged grass have been reported for another harvester termite, the widespread *Hodotermes mossambicus*, but the depots were small chambers around the nest perimeter or small chambers near the surface, rather than being part of the foraging tunnels (Hartwig 1963, 1965; Coaton and Sheasby 1975). Probably, this cache system evolved independently, as these species belong to different subfamilies and the depots have different structures. However, Darlington (1982) described and quantified depots along the foraging tunnels of *M. michaelsoni* and the Brazilian *Syntermes molestus*. The depots of *M. michaelsoni* were especially similar to those of *B. hainesi*, and Darlington speculates that termites foraging on dispersed food such as grass or litter ought to evolve tunnel system with caches because foraging must occur in episodes. She calculates that the volume of caches underlying an area was similar to the volume of forage gathered in that area in one night. The presence of caches of grass pieces in the depots of *B. hainesi* strongly suggests that the workers that harvest the grass on the surface are distinct from the tunnel transport workers, and that the system is to some degree a “bucketbrigade,” a system of greater efficiency than one in which each individual harvests and transports each piece of grass all the way to the nest. Leafcutter ants also use caching and “bucket-brigade” transport for leaf pieces (Hart and Ratnieks 2001; Anderson et al. 2002), thus partitioning the task of foraging into cutting, caching and multiple transporting stages. Caching was more likely when traffic was heavy or bottlenecked, and incurred the cost of mismatching the leaf piece with the size of the subsequent transporting worker, thus slowing transport. Anderson et al. (2002) used simulations to test for optimality in such transport systems. It is likely that *B. hainesi* also tends to cache more grass pieces when cutting rate exceeds transport. The partitioning of foraging in this manner unlinks harvesting, a mostly nocturnal task which carries the risk of exposure to desiccation and predation, from transport, which is relatively safe within the tunnel system and can probably proceed more or less around the clock, as it does in *M. michaelsoni*. The obvious advantage of such a system may underlie the reason it has evolved in such diverse taxa as ants and termites (and humans).

The results leave the spatial extent and size of the colony of this termite undetermined. We found no primary reproductives in the dissected nest and no structure that might be a “royal cell.” Combined with the fact that at least two live nests only about 6 meters apart were connected with tunnels suggests that a colony may consist of multiple nests, some possibly deep in the ground, as suggested by the existence of tunnels-to-depth. Fuller (1915, as cited in Lee and Wood 1971) reported that adjacent mounds of *Trinervitermes trinervoides* were interconnected through subterranean tunnels. On the other hand, Darlington (1982) found the remains of dead soldiers and workers in the contact zone between foraging tunnel systems of neighboring mounds of *M. michaelsoni*, suggesting the occurrence of territorial battles.

Ebeling and Pence (1957) described how *Reticulitermes hesperus* use fine soil particles mixed with saliva to line their tunnels. In light of the extreme aridity of the Namib Desert, and the fact that nest and tunnel construction require water, it seems inescapable that the termites have access to moist soil, probably at great depth. When first brought to the surface and dumped, soil excavated by *H. mossambicus* in the study area was damp (personal observation), yet no trace of dampness was detectable even in excavations over 2 m deep. Yakushev (1968, as cited in Lee and Woods 1971) reports that some termite species may make tunnels to moisture as deep as 70 m.

Photographs included in Hill's (1942) treatise of Australian termites show that *Coptotermes acinicaformis* and *C. lacteus*, both mound-builders, construct nests with a very thick “carapace,” much like *B. hainesi*. This feature is lacking in the other species examined in Hill's book. In contrast to the nests of *B. hainesi*, the nests of subterranean-nesting termites are often surrounded by an empty space rather than a “carapace” (Noirot 1970). Perhaps the difference lies in the relative instability of the dry sands in which *B. hainesi* nests.

This estimate of the nest population is surely an underestimate of the actual population, for it is likely that a substantial fraction of the termites were in the foraging tunnels at the time of collection. Even after removal of the nest, abundant termites were found in the tunnels during several days of excavation. Whether their home was in the collected nest or in another, possibly a deeper nest, could not be determined.

Considering the density of foraging access points as well as the biomass of termites and the amount of grass pieces found in the nest and tunnels, it is likely that *B. hainesi* foraging has a considerable impact on the sparse grasslands of the eastern Namib Desert. This is more likely because conditions conducive to the growth of grasses may occur less than annually, and then only for short periods. Estimates for grass consumption in a “saturated” population of *H. mossambicus* in a more lush habitat (Zululand) ranged up to 1 to 3 metric tons per ha, practically the total yield of hay, but other estimates were much lower (Coaton and Sheasby 1975). There are many reports of *H. mossambicus* creating bare spots through their harvesting activity. It has been suggested that this termite is the cause of the fairy circles mentioned in the Materials and Methods (Becker 2007), but this claim is contested (van Rooyen et al. 2004). Darlington (1982) estimated the nightly forage collected by *M. michealsoni* to be approximately 0.6 to 1.1 kg. Finally, Darlington (1982) showed that the surface access points in *M. michaelsoni* tunnels to be dense enough that termites need rarely travel more than 10 cm from an opening to forage. The actual density of access points in *B. hainesi* is unknown, but is clearly higher than indicated in Figures 14 and 15, because many of the crossconnecting passages were left unexcavated. Likewise, Darlington (1982) estimated that the nest of *M. michaelsoni* has a total of 6 km of permanent foraging tunnels, but in view of unexcavated cross-passages and the difficulty of placing colony boundaries on *B. hainesi*, a corresponding estimate is undetermined for this study. *B. hainesi* colonies are much smaller than those of *M. michealsoni*, yet their work is still impressive. It is likely that similar tunneland- depot systems are characteristic of many harvesting termites.
